# Neurosyphilis presenting with Guillain–Barre syndrome: a case report

**DOI:** 10.1186/s12883-023-03471-5

**Published:** 2023-11-25

**Authors:** Hoameng Ung, Dominic Ferrey

**Affiliations:** Department of Neurosciences, University of California San Diego, San Diego, California USA

**Keywords:** Syphilis, Guillain–Barre Syndrome, Acute inflammatory demyelinating polyneuropathy, Case report

## Abstract

**Background:**

Syphilis is associated with a wide variety of systemic presentations, earning it the moniker “The great mimicker”. Neurosyphilis is classically associated with meningovasculitis in the acute-subacute stage and tabes dorsalis and dementia paralytica in later stages. However, one of the less well described presentations include Guillain–Barre Syndrome. This case presents a patient with an ascending polyneuropathy suspicious for Guillain–Barre Syndrome who also had other atypical findings including a truncal sensory loss, optic disc swelling, and rash ultimately found to have neurosyphilis. Electrodiagnostic testing was consistent with demyelination, supporting a diagnosis of neurosyphilis associated Guillain–Barre Syndrome.

**Case presentation:**

A 37-year-old female presented to the emergency department with a weakness and difficulty swallowing. She described a three-month history of symptoms, initially starting with a persistent headache followed by one month of a pruritic rash on her chest, palms, and soles. Two weeks prior to presentation, she developed progressive weakness in her arms, numbness in her arms and chest, and difficulty swallowing. Neurological exam was notable for multiple cranial neuropathies, distal predominant weakness in all extremities, length-dependent sensory loss, and hyporeflexia. Investigation revealed a positive Venereal Disease Research Laboratory in her cerebrospinal fluid without significant pleocytosis, contrast enhancement in cranial nerves V, VII, and VIII on MRI, and a demyelinating polyneuropathy on electrodiagnostic testing. She was diagnosed with Guillain–Barre syndrome, secondary to neurosyphilis. The patient acutely declined and required intubation, and ultimately made a full recovery after treatment with plasmapheresis and penicillin.

**Conclusions:**

This case describes a clinical entity of syphilitic Guillain–Barre Syndrome and highlights the importance of including syphilis in the differential of any patient presenting with ascending polyradiculopathy, especially given the resurgence of syphilis.

## Background

The prevalence and incidence of syphilis in the United States has reached new highs since the 1940s, rising by 164% and 175% respectively between 2008 and 2018 [1, 2]. Typical manifestations of neurosyphilis can be divided into early and late stages, with the former encompassing meningitis and meningovasculitis, and the latter manifesting as tabes dorsalis and dementia paralytica [3]. Syphilis has also been associated with many atypical presentations including those that mimic idiopathic intracranial hypertension and Parkinson’s Disease [4]. It is thus increasingly important to recognize the myriad of clinical manifestations of syphilis due to the devastating sequelae if left undiagnosed and untreated. Here, we describe a case of neurosyphilis that presented with clinical Guillain–Barre Syndrome (GBS). While several case reports have described GBS-like symptoms in a patient with syphilis, this case is, to our knowledge, the first electrodiagnostically confirmed case of syphilitic GBS without coexisting confounders.

## Case presentation

**Fig. 1:**
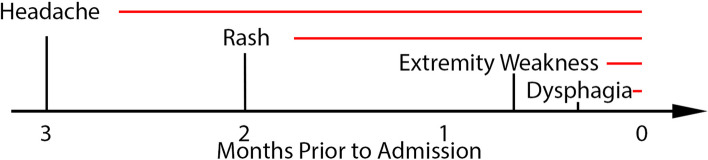
Timeline of symptom presentation

A previously healthy 37-year-old female presented to the emergency department with headache, rash, and bulbar weakness. Three months prior to admission (PTA), she gradually developed a constant, severe headache with photophobia. She presented to an urgent care clinic for these symptoms and was prescribed symptomatic medications without resolution. Two months PTA, she developed a pruritic rash on her chest, neck, palms, and soles. She again presented to urgent care and was given antihistamines for the rash. Two weeks PTA, she began to feel weakness in her arms as well as numbness in her arms, chest, and thighs. When her weakness progressed and she started to have difficulty swallowing, she came to the emergency department. She denied any fevers but reported intermittent chills for the past three months (Fig. 1).

### Examination

**Fig. 2:**
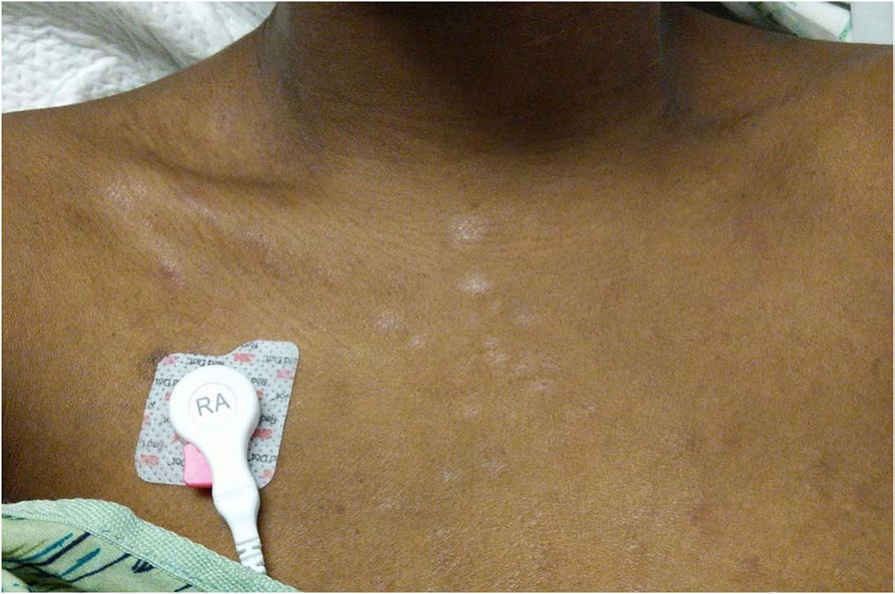
Thin scaly papules on chest. There are mixed scaly and erythematous papules on the neck as well as hands and soles (not pictured)

The examination was notable for bilateral cranial nerve V, VI and VII palsies. Bilateral optic nerve head edema was seen on fundoscopic exam. Near visual acuity was 20/20 and color vision was intact in both eyes. Pupils were equal, 3 mm and equally reactive without RAPD. The motor exam showed weakness in neck flexion (4/5) as well as distal greater than proximal bilateral upper extremity weakness (3/5). Reflexes were absent throughout and there was no Babinski sign. Sensation in her extremities was significantly decreased to all modalities in a stocking glove distribution up to her knees and forearms. She also noted decreased sensation below her neck to her waist, circumferentially, though it did not follow a clear spinal dermatome. The skin exam was notable for multiple morphologies including thin scaly papules on the upper chest and neck with more erythematous papules on the neck, back, flanks and extremities. Faint erythematous macules were present on her palms and soles (Fig. 2).

The patient’s presentation thus far of weakness, numbness, and areflexia were suggestive of a polyradiculopathy, such as that as seen in GBS. However, the patient’s history of headache, and exam findings of optic nerve head edema and skin rash were atypical. This prompted a broad differential and necessitated further imaging, infectious, and rheumatologic workup.

### Diagnostic investigation

**Fig. 3:**
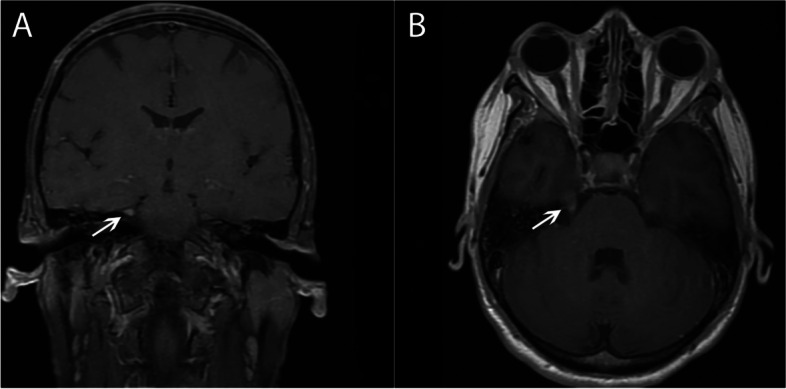
T1-post contrast coronal (**A**) and axial (**B**) MRI demonstrating bilateral (right greater than left) cranial nerve V enhancement

MR head imaging was obtained that was remarkable for contrast enhancement of cranial nerves V, VII, and VIII bilaterally (Fig. 3).

MRI’s of the whole spine with and without contrast was obtained that was only notable for mild C5-6 spinal canal stenosis. Lumbar puncture was significant for an elevated opening pressure of 36 cmH20 (normal: 6–25 cmH20) and cerebrospinal fluid (CSF) analysis revealed an RBC < 1 /mm3 (0), slight pleocytosis with WBC of 6 /mm3 (0–5 /mm3), protein of 46 mg/dL (15–45 mg/dL), and glucose of 63 mg/dL (40–70 mg/dL). An infectious workup consisting of a CSF Infectious PCR panel for 14 bacteria, viruses, and yeast such as N. meningitidis, HSV 1/2 and Cryptococcus, as well as an HIV PCR, was negative. Serologies for Coxsackievirus, campylobacter jejuni were negative. Anti-ganglioside antibodies for Miller-Fisher syndrome were negative. Rheumatologic and systemic inflammatory etiologies that may present with skin findings, such as ANCA vasculitis and sarcoidosis, were also considered, but screening labs including Angiotensin-Converting Enzyme levels, ANA, ANCA, and SSA/B were unremarkable. However, serum RPR was positive in 1:128 titer, and CSF VDRL was positive with 1:1 titer, confirming neurosyphilis.

**Fig. 4:**
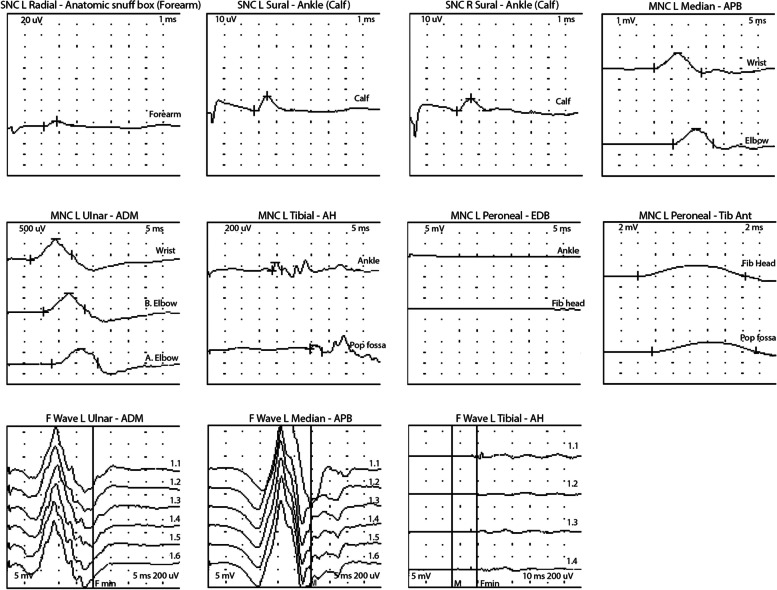
Motor and sensory nerve conduction study waveforms. SNC = Sensory Nerve Conduction. MNC = Motor nerve conduction. APB = Abductor Pollicis Brevis, ADM = Abductor Digiti Minimi, EDB = Extensor Digitorum Brevis, AH = Adductor Hallicus

As GBS is not classically thought of as a presentation of syphilis, further diagnostics with inpatient electromyography (EMG) and a nerve conduction study (NCS) were also performed. NCS of the left arm and leg showed low amplitude motor studies as well as prolonged distal motor latencies and slowing of conduction velocity. F-waves in the ulnar, median, and tibial nerves were absent (Table 1) (Fig. 4). Needle EMG of the selected muscles of the left upper and lower extremities showed decreased recruitment of normal amplitude and normal duration MUAPs. There was no abnormal spontaneous activity (Table 2).

**Table 1:** Selected NCS findings

Sensory Nerve Conduction					
**Nerve/Sites**	**Onset Latency (ms)**	**Peak Latency (ms)**	**Amplitude (uV)**	**Distance (mm)**	**Velocity (m/s)**
**Left Sural—Ankle**					
Calf	2.7	3.5	14	140	51.3
**Right Sural—Ankle**					
Calf	2.7	3.5	12	140	51.7
**Left Radial—Anatomical snuff box**					
Forearm	2.1	2.8	10	100	47.5
Motor Nerve Conduction					
**Nerve/Sites**	**Latency (ms)**		**Amplitude (mV)**	**Duration (ms)**	**Velocity (m/s)**
**Left Median—APB**					
Wrist	15.1 (< 4.4)		1.4 (> 4.0)	13.4	
Elbow	20.3		1.4	11.7	41.9 (> 49)
**Left Ulnar—ADM**					
Wrist	6.8 (< 3.6)		1.0 (> 5.0)	12	
Below Elbow	10.6		0.9	11.7	47.2 (> 49)
Above Elbow	12.9		0.7	13.4	44.9
**Left Peroneal—EDB—No Response**					
**Left Tibial—AH**					
Ankle	14.9 (< 6.0)		0.1 (> 3.0)	6.5	
Popliteal Fossa	29.5		0.1	3.6	21.9 (> 39)
F-wave					
**Nerve—Muscle**	**F Latency (ms)**		**M Latency (ms)**	**F-M Latency (ms)**	
Left Ulnar—ADM	NR				
Left Median—APB	NR		NR	NR	
Left Tibial—AH	NR		NR	NR	

*APB* Abductor Pollicis Brevis, *ADM* Abductor Digit Minimi, *EDB* Extensor Digitorum Brevis, *AH* Adductor Hallicus, *NR* No Response. EMG in the left tibialis anterior, vastus lateralis, and first dorsal interosseous muscles showed moderately decreased recruitment. Normal values are shown in parenthesis

**Table 2:** Needle EMG findings

	**Spontaneous Activity**					**Voluntary Actvity**				**Comment**
Muscle	**IA**	**Fib**	**PSW**	**Fasc**	**Misc**	**Amp**	**Dur**	**PPP**	**Recruitment**	**Pattern**
L. Tibialis anterior	Normal	None	None	None	None	Normal	Normal	Normal	Mod Decr	Neuropathic
L. Vastus lateralis	Normal	None	None	None	None	Normal	Normal	Normal	Mod Decr	Neuropathic
L. First dorsal interosseous	Normal	None	None	None	None	Normal	Normal	Normal	Mod Decr	Neuropathic

*Abbreviations*: *IA* Insertional activity, *Fib* fibrillations, *PSW* polyspike wave, *Fasc* Fasciculations, *Amp* Amplitude, *Dur* Duration, *PPP* polyphasic potentials, *Mod* Moderately, *Decr* decreased

These findings confirmed a mixed demyelinating polyneuropathy predominantly affecting motor nerves consistent with an acute inflammatory demyelinating polyneuropathy variant of GBS. The clinical syndrome as well as electrodiagnostic testing provided support for treatment of both GBS as well as neurosyphilis.

### Clinical course

The patient’s weakness initially progressed and negative inspiratory force was -15 cmH2O. She was electively intubated for airway protection. Plasma exchange was started every other day for five days as treatment of the GBS and penicillin was also started for treatment of the neurosyphilis. She was extubated on day four and continued to recover. She was eventually discharged home on day 18 after completion of 14 days of IV penicillin therapy. The patient achieved almost complete recovery by day of discharge, though unfortunately is currently lost to follow up.

## Discussion and conclusions

In our case, the patient did have classic symptoms of neurosyphilis, including a headache and cranial neuropathies, as well as symptoms consistent with GBS. As she described a three month history of new headache, and since secondary syphilis typically occurs two to eight weeks after inoculation, we suspect that she developed neurosyphilis early into her infection [5]. It is often mistaken that neurosyphilis is a later stage of syphilis, when in fact inoculation of the central nervous system can occur within days of primary infection [3]. An estimated 25% of syphilis result in neurosyphilis and may progress in several stages. In the early stages, one may be asymptomatic with laboratory findings characterized by reactive serum and CSF VDRL tests. Meningovasculitis can present as well, resulting in strokes and meningomyelitis. If left untreated, the later stages of neurosyphilis consist of general paresis with progressive dementia (dementia paralytica) as well as tabes dorsalis. It has been postulated that the degree of CSF pleocytosis and hypoglycorrhachia is correlated with the severity of symptoms and stages, with an early stage of neurosyphilis associated with mild pleocytosis, acute meningeal neurosyphilis with significant pleocytosis and decreased glucose, and chronic disease associated with normal CSF findings [6–9]. Wang et al. reported a median CSF WBC of 5.5 /mm3, with a range of (0–145.2 /mm3) in 103 patients with neurosyphilis [8]. Similarly, Pastuszczak et al. described a correlation of CSF pleocytosis and decreased CSF glucose with VDRL reactivity [9]. In this case, the minimal pleocytosis and normal glucose was unusual given the severity of her symptoms, but it is possible that the neurosyphilitic component of her presentation is in the earlier stages, and that GBS, which is not typically associated with significant pleocytosis or hypoglycorrhachia, may be the primary driver of her presenting symptoms.

Guillain–Barre syndrome (GBS) is an immune-mediated polyneuropathy that typically presents with a monophasic ascending pattern of sensory loss and weakness, initially in the hands and feet. In two-thirds of cases, there is an antecedent infection. Examination is notable for hypo- or areflexia, in addition to weakness and sensory loss, in a stocking glove distribution. While GBS commonly presents as a primarily demyelinating form, other variants have been described such as a motor-axonal neuropathy and Miller-Fisher syndrome, a primarily axonal neuropathy that presents with ophthalmoplegia, areflexia, and ataxia [10]. In this case, the nerve conduction testing demonstrated prolonged latencies in multiple motor nerves as well as absent F waves, consistent with demyelination. These nerve conduction findings overall support an acute inflammatory demyelinating polyradiculoneuropathy variant of GBS. It is important to note that cranial neuropathies have been described in syphilitic meningitis, though extremity involvement favors GBS [11].

There have been several studies describing GBS-like symptoms in a patient with syphilis. In 1998, Stepper et al. described a case of a 36-year-old man who presented with ataxia, diplopia, and a left cranial nerve VI palsy that improved with IVIG, but later relapsed and was then diagnosed with neurosyphilis that improved with penicillin. Byrne in 1990 describes a syphilitic paraparesis in a 34-year-old woman who presented with ataxia and secondary syphilis that improved with penicillin and in 2011, Wasserman described a case of a 40-year-old man who presented difficulty walking, facial asymmetry, and numbness in lower legs with a positive VDRL that was also treated successfully with penicillin. In these cases, electrodiagnostics were not consistent with GBS or were not performed [12–14]. More recently McNiel and Berger describes cases of suspected GBS from syphilis, though each are confounded by other infections including a preceding upper respiratory infection, COVID, or COVID vaccination [15, 16]. In each of the above cases, it remains unclear whether the patient’s polyradiculopathy was demyelinating in nature, or even whether syphilis was the primary inciting factor.

In summary, we believe this case of clinical and electrodiagnostic GBS in the setting of neurosyphilis without other preceding confounders strongly suggests syphilis as the underlying etiology. While penicillin is the mainstay of treatment, it remains unclear whether immunotherapy (immunoglobulins or plasmapheresis) is necessary. This case reiterates the need to include syphilis in the differential of any patient presenting with an acute ascending paralysis concerning for GBS, especially if there is an atypical presentation such as papilledema. This is the case regardless if initial CSF profile is relatively benign, and highlights the need to understand the variety of syphilis presentations as the incidence increases.

## Data Availability

Data sharing is not applicable to this article as no datasets were generated or analysed during the current study.

## Abbreviations

GBS: Guillain–Barre Syndrome
PTA: Prior to admission
EMG: Electromyography
NCS: Nerve conduction study
CSF: Cerebrospinal fluid
